# Prevalence of 3 Healthy Lifestyle Behaviors Among US Adults With and Without History of Stroke

**DOI:** 10.5888/pcd16.180409

**Published:** 2019-02-21

**Authors:** Ryan R. Bailey, Allison Phad, Ryan McGrath, Rachel Tabak, Debra Haire-Joshu

**Affiliations:** 1 Washington University in St Louis, St Louis, Missouri; 2 North Dakota State University, Fargo, North Dakota

## Abstract

**Introduction:**

Engaging in healthy lifestyle behaviors decreases risk for cardiometabolic complications, which is of particular concern for stroke survivors whose history of stroke (HOS) increases cardiometabolic risk. Population-based estimates of healthy behaviors in adults with HOS are lacking but could be used to inform research, policy, and health care practice. The objective of this study was to calculate and compare population-based estimates of the prevalence of consuming 1 or more fruit and 1 or more vegetable daily, meeting weekly aerobic physical activity recommendations, having a body mass index (BMI) of less than 25 kg/m^2^, and the number of healthy behaviors among US adults with and without HOS.

**Methods:**

We used data from the 2015 Behavioral Risk Factor Surveillance System. Weighted and age-adjusted (to the 2000 US standard population) prevalence estimates and adjusted odds ratios (AORs, adjusted for demographic variables) were computed for study variables.

**Results:**

Adults with HOS were less likely than adults without HOS to consume 1 or more fruit and 1 or more vegetable daily (AOR = 0.85; 95% confidence interval [CI], 0.79–0.91), meet weekly aerobic physical activity recommendations (AOR = 0.72; 95% CI, 0.67–0.78), and engage in 2 (AOR = 0.86; 95% CI, 0.79–0.94) or 3 (AOR = 0.73; 95% CI, 0.64–0.82) healthy behaviors. Adults with HOS were more likely to engage in 0 healthy behaviors (AOR = 1.26; 95% CI, 1.16–1.37). Having a BMI of less than 25 kg/m^2^ and engaging in 1 healthy behavior were similar between groups.

**Conclusion:**

Prevalence of individual and total number of healthy behaviors was lower in adults with HOS for several healthy behaviors. Future research, policy, and health care practice is needed to promote healthy behaviors in adults with HOS.

**Summary**

**What is already known on this topic?**

Healthy lifestyle behaviors decrease risk for cardiometabolic conditions and recurrent stroke for adults with history of stroke.

**What is added by this report?**

This study updates and compares population-based estimates for 3 lifestyle behaviors — consuming 1 or more fruit and 1 or more vegetable daily, meeting weekly aerobic physical activity recommendations, and having a body mass index of less than 25 kg/m^2^ — among adults with and without history of stroke.

**What are the implications for public health practice?**

Results indicate that adults with history of stroke report low fruit and vegetable consumption and physical activity, suggesting that additional health behavior interventions are needed.

## Introduction

Healthy lifestyle behaviors, including fruit and vegetable consumption, physical activity, and having a healthy weight, protect against many chronic conditions, including cancer, cardiovascular disease, diabetes, and stroke (1). Lifestyle modification interventions have demonstrated that lifestyle behavior modification can decrease cardiometabolic risk through improved blood pressure, blood cholesterol, triglycerides, blood glucose, and body weight (2–5). Improvements in these factors are pertinent to persons with history of stroke (HOS), because HOS increases risk for diabetes, recurrent stroke, and cardiovascular mortality (6,7).

Common goals of lifestyle programs include weight loss through modification of dietary intake and physical activity. Weight loss of approximately 5% is sufficient to achieve improvement in cardiometabolic indices of health (eg, blood pressure, dyslipidemia, diabetes) (8). However, weight loss interventions among people with HOS are lacking even though weight loss is recommended for managing cardiometabolic risk factors common among people with HOS (8). Health promotion interventions in stroke survivors have demonstrated favorable improvements in both body weight and cardiometabolic risk (9,10). Epidemiological investigation has also demonstrated a dose–response relationship where engaging in an increased number of healthy behaviors is associated with decreased all-cause and cardiovascular mortality in people with HOS (11).

Despite scientific evidence and clinical guidelines (6) that promote participation in healthy lifestyle behaviors after stroke, population-based estimates of the prevalence of healthy behaviors among people with HOS are lacking. A better understanding of such behaviors can inform future research, federal policy, and the development of lifestyle behavior modification interventions tailored for people with HOS. Therefore, the purpose of this study was to compare the prevalence of 3 healthy lifestyle behaviors (consuming fruits and vegetables, engaging in physical activity, and having a body mass index [BMI] <25 kg/m^2^) and the total number of healthy behaviors among US adults with and without HOS.

## Methods

### Study population

In this cross-sectional study, we analyzed data from the 2015 Behavioral Risk Factor Surveillance System (BRFSS) to examine healthy behaviors among adults with HOS, using adults without HOS as the reference group. The BRFSS is an ongoing, state-based, telephone survey that uses randomly selected landline and cellular telephone numbers to monitor behavioral risk factors among noninstitutionalized adults aged 18 years or older, and it includes data from all 50 states, the District of Columbia, Puerto Rico, and Guam. Median response rates in 2015 were 48.2% for landline and 47.2% for cellular telephone surveys, which are comparable to response rates for other national telephone-based surveys. More information about the 2015 BRFSS is available (www.cdc.gov/brfss/annual_data/annual_2015.html).

Respondents were identified as having HOS by their response to the following BRFSS question: “Has a doctor, nurse, or other health professional ever told you that you had a stroke?” Of 441,456 respondents in the 2015 BRFSS, 1,290 individuals responded “don’t know/not sure” or refused to answer and were excluded from analysis. This resulted in a study population of 440,166 respondents (HOS, n = 18,269; no HOS, n = 421,897).

### Demographic characteristics and health conditions

Demographic characteristics were sex (male/female), age in years (18–24, 25–44, 45–64, ≥65), race/ethnicity (non-Hispanic white, non-Hispanic black, Hispanic, other), marital status (married or part of an unmarried couple, previously married, never married), education (some high school, graduated high school, some college, graduated college), and annual household income (<$15,000, $15,000 to <$25,000, $25,000 to <$35,000, $35,000 to <$50,000, ≥$50,000). Health conditions were presence of hypertension, high cholesterol, and diabetes. These health conditions were determined by respondents’ responses to “Has a doctor, nurse, or other health professional ever told you that you had [condition]?” where the question was asked for each health condition.

### Study variables

The 3 healthy behaviors assessed were self-reported fruit and vegetable consumption, self-reported physical activity, and having a BMI of less than 25 kg/m^2^, which was calculated by using self-reported weight and height at the time of survey. BRFSS tracks fruit and vegetable consumption as a marker of nutritional intake in lieu of performing a comprehensive nutritional assessment. Respondents were asked to indicate how many times per day, week, or month (during the previous month) they consumed fruit, 100% fruit juice, beans, and vegetables. BRFSS identifies individuals who consume 1 or more fruit and 1 or more vegetable daily, which was defined as a healthy behavior in this study, but does not track whether individuals meet national recommendations for fruit and vegetable consumption because recommendations are based on an individual’s age, sex, and physical activity level rather than a specific number of servings (12). BRFSS also asks respondents to provide information on moderate- and vigorous-intensity physical activity other than regular job duties during the previous month, including exercise, leisure, and household physical activity. Meeting recommended levels of weekly aerobic physical activity was defined as accruing at least 150 minutes of moderate-intensity physical activity per week, at least 75 minutes of vigorous-intensity physical activity per week, or an equivalent combination of moderate- and vigorous-intensity physical activity per week (13). Lastly, having a BMI less than 25 kg/m^2^ was selected, because it is associated with decreased risk for chronic disease and all-cause mortality (14). We also computed the total number of healthy behaviors for each participant to examine clustering of healthy behaviors.

### Data analysis

We used SAS for Windows, Version 9.4 (SAS Institute Inc) to analyze data and to account for the complex sampling design, including selection probability and survey nonresponse. Age-adjusted (to the 2000 US standard population) and weighted prevalence estimates with 95% confidence intervals (CIs) were calculated for demographic characteristics, health conditions, and study variables. For number of healthy behaviors, only respondents who provided data for all 3 healthy behaviors were analyzed. To examine the relationship of individual and number of healthy behaviors by HOS status, we used logistic regression to compute unadjusted odds ratios (ORs) with HOS status as a predictor in the model. Adjusted ORs, controlling for demographic variables (ie, sex, age, race/ethnicity, marital status, education, and annual household income) were also calculated. *P* values were not reported because most variables were significant as a result of the large sample size obtained when we weighted the data (15), but significance can be inferred by examining overlap of 95% CIs (16).

## Results

Most respondents were female, aged 45 to 65 years, non-Hispanic white, married or part of an unmarried couple, had attended college, and had an annual household income of $50,000 or more (Table 1). Respondents with HOS were older and had lower income than respondents with no HOS. Most respondents with HOS were aged 65 years or older and had an annual household income of $15,000 to less than $25,000. In contrast, most respondents without HOS were aged 25 to 44 years and had an annual household income of $50,000 or more. Respondents with HOS had a higher prevalence of hypertension, high cholesterol, and diabetes than respondents with no HOS. 

**Table 1:** Demographic Characteristics and Health Conditions Among US Adults, by History of Stroke Status^a^, Behavioral Risk Factor Surveillance System, 2015

Characteristic	Number of Respondents^b^	Total	History of Stroke (n = 18,269)	No History of Stroke (n = 421,897)
		% (95% Confidence Interval)^c^		
**Sex**				
Male	186,362	48.9 (48.6–49.2)	46.4 (43.3–49.4)	48.9 (48.6–49.2)
Female	253,804	51.1 (50.8–51.4)	53.6 (50.6–56.7)	51.1 (50.8–51.5)
**Age, y**				
18–24	24,172	12.8 (12.6–13.1)	1.2 (0.8–1.6)	13.2 (12.9–13.4)
25–44	93,386	33.8 (33.5–34.0)	10.4 (9.4–11.4)	34.5 (34.2–34.8)
45–64	170,046	33.9 (33.6–34.2)	39.0 (37.6–40.5)	33.7 (33.5–34.0)
≥65	152,562	19.6 (19.4–19.7)	49.3 (47.9–50.7)	18.6 (18.4–18.8)
**Race/ethnicity**				
Non-Hispanic white	335,144	61.5 (61.2–61.8)	58.2 (55.1–61.3)	61.6 (61.3–61.9)
Non-Hispanic black	34,267	11.7 (11.5–11.9)	18.1 (15.6–20.7)	11.5 (11.3–11.8)
Hispanic	35,688	17.1 (16.8–17.3)	13.3 (10.8–15.8)	17.1 (16.9–17.4)
Other	27,715	8.1 (7.9–8.3)	8.4 (6.6–10.2)	8.1 (7.9–8.3)
**Marital status**				
Married or unmarried couple	245,327	55.1 (54.8–55.3)	41.5 (39.1–43.8)	55.4 (55.1–55.7)
Previously married	124,305	19.0 (18.8–19.2)	31.0 (28.6–33.5)	18.7 (18.5–18.9)
Never married	67,503	25.2 (25.0–25.5)	26.6 (24.1–29.2)	25.2 (25.0–25.4)
**Education**				
Some high school	34,065	14.2 (13.9–14.5)	23.4 (20.6–26.1)	13.9 (13.7–14.2)
Graduated from high school	122,846	27.9 (27.6–28.1)	29.8 (27.2–32.3)	27.8 (27.5–28.1)
Some college	120,185	30.9 (30.6–31.2)	33.3 (30.4–36.2)	30.9 (30.6–31.2)
Graduated from college	161,311	26.6 (26.4–26.9)	13.2 (11.8–14.6)	27.0 (26.7–27.2)
**Annual household income, $**				
<15,000	37,812	9.5 (9.3–9.7)	20.1 (17.7–22.5)	9.2 (9.1–9.4)
15,000 to <25,000	58,976	14.1 (13.9–14.3)	23.7 (20.9–26.5)	13.9 (13.7–14.1)
25,000 to <35,000	39,138	8.7 (8.5–8.9)	10.1 (8.4–11.9)	8.7 (8.5–8.8)
35,000 to <50,000	51,949	11.3 (11.1–11.5)	8.85 (7.4–10.3)	11.3 (11.1–11.5)
≥50,000	173,229	39.2 (38.9–39.5)	19.6 (17.1–22.1)	39.8 (39.5–40.1)
**Hypertension^a^**	177,434	29.9 (29.6–30.1)	58.1 (55.0–61.1)	29.1 (28.9–29.4)
**High cholesterol^a^**	159,369	31.5 (31.2–31.8)	49.4 (45.8–53.0)	31.1 (30.8–31.4)
**Diabetes^a^**	56,997	9.6 (9.4–9.7)	21.4 (19.7–23.2)	9.2 (9.0–9.4)

a History of stroke, hypertension, high cholesterol, and diabetes were determined by respondents’ response to “Has a doctor, nurse, or other health professional ever told you that that you had [condition]?” where the question was asked for each health condition.

b Unweighted number of respondents. For demographic characteristics, categories may not sum to survey total because some respondents did not respond to all survey questions. For health characteristics, the number of respondents with the characteristic is reported.

c Estimates are weighted and age-adjusted to the 2000 US standard population, except for age groups, which display age group–specific percentages. Categories may not sum to 100% because some respondents did not respond to all survey questions.

The prevalence of 2 healthy behaviors — consuming 1 or more fruit and 1 or more vegetable daily and meeting weekly aerobic physical activity recommendations — was approximately 50% among respondents as a whole (Table 2). However, prevalence for these 2 healthy behaviors was lower among respondents with HOS than respondents without HOS — 45.2% versus 52.0% for fruit and vegetable consumption and 42.5% versus 50.9% for meeting weekly physical activity recommendations. Approximately one-third of respondents had a BMI of less than 25 kg/m^2^, regardless of HOS status. 

**Table 2:** Prevalence of 3 Healthy Behaviors Among US Adults, by History of Stroke Status, Behavioral Risk Factor Surveillance System, 2015

Healthy Behavior	Number of Respondents^a^	Total	History of Stroke (n = 18,269)	No History of Stroke (n = 421,897)
		% (95% Confidence Interval)^b^		
Consumes ≥1 fruit and ≥1 vegetable daily^c^	381,649	51.8 (51.5–52.1)	45.2 (41.9–48.6)	52.0 (51.7–52.4)
Meets weekly aerobic PA recommendations^d^	387,150	50.6 (50.2–50.9)	42.5 (39.2–45.9)	50.9 (50.6–51.2)
Has a BMI <25 kg/m^2e^	403,977	35.8 (35.5–36.1)	33.0 (29.9–36.1)	35.9 (35.6–36.2)

Abbreviations: BMI, body mass index; PA, physical activity.

a Unweighted number of respondents. The number of respondents with each healthy behavior is reported.

b Estimates are weighted and age-adjusted to the 2000 US standard population.

c Respondents were asked to indicate how many times per day, week, or month (during the previous month) they consumed fruit, 100% fruit juice, beans, and vegetables.

d Weekly aerobic physical activity recommendations include ≥150 min of moderate-intensity PA per week, ≥75 min of vigorous-intensity PA per week, or an equivalent combination of moderate- and vigorous-intensity PA per week.

e BMI (kg/m^2^) was calculated using self-reported weight and height at the time of survey.

Before adjustment, respondents with HOS had lower odds of engaging in each healthy behavior compared with respondents without HOS (Figure). After adjustment for demographic characteristics, only the difference in having a BMI <25 kg/m^2^ between groups was attenuated.

**Figure:**
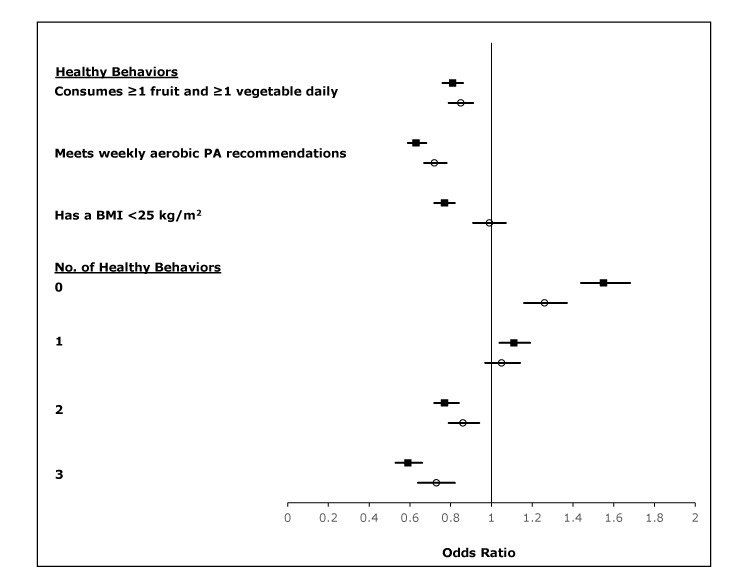
Crude and adjusted odds of individual and total number of healthy behaviors in adults with history of stroke (HOS), 2015 Behavioral Risk Factor Surveillance System. Adults without HOS was the reference group. Sex, age, race/ethnicity, marital status, education, and annual household income were controlled for in the adjusted odds. Black squares represent crude values, and open circles represent adjusted values; horizontal bars represent 95% confidence intervals.

**Table:** 

Category	Crude	Adjusted
	Odds Ratio (95% Confidence Interval)	
**Healthy behavior**		
Consumes ≥1 fruit and ≥1 vegetable daily	0.81 (0.76–0.86)	0.85 (0.79–0.91)
Meets weekly aerobic physical activity recommendations	0.63 (0.59–0.68)	0.72 (0.67–0.78)
Has a body mass index <25 kg/m^2^	0.77 (0.72–0.82)	0.99 (0.91–1.07)
**No. of healthy behaviors**		
0	1.55 (1.44–1.68)	1.26 (1.16–1.37)
1	1.11 (1.04–1.19)	1.05 (0.97–1.14)
2	0.77 (0.72–0.84)	0.86 (0.79–0.94)
3	0.59 (0.53–0.66)	0.73 (0.64–0.82)

Data for all 3 healthy behaviors were available from 346,930 respondents **(**HOS, n = 13,917; no HOS: n = 333,013**)** for analysis. The age-adjusted, estimated prevalence of number of healthy behaviors indicated that respondents with HOS engaged in fewer healthy behaviors than did respondents without HOS (Table 3). The prevalence of (or the proportion of) not engaging in any healthy behavior was higher (24.8% vs 18.8%) and the prevalence of engaging in 3 healthy behaviors was lower (8.1% vs 12.7%) in respondents with HOS compared with respondents without HOS. Similarly, the odds of not engaging in any healthy behavior were higher in respondents with HOS compared with respondents without HOS (OR = 1.26; 95% CI, 1.16–1.37), while odds of engaging in 2 (OR = 0.86; 95% CI, 0.79–0.94) or 3 (OR = 0.73; 95% CI, 0.64–0.82) healthy behaviors were lower, even after adjusting for demographic characteristics (Figure).

**Table 3:** Prevalence of Number of Healthy Behaviors Among US Adults, by History of Stroke Status, Behavioral Risk Factor Surveillance System, 2015

Number of Healthy Behaviors^a^	Number of Respondents^b^	Total	History of Stroke (n = 13,917)	No History of Stroke (n = 333,013)
		% (95% Confidence Interval)^c^		
0	63,895	19.0 (18.8–19.3)	24.8 (22.2–27.5)	18.8 (18.6–19.1)
1	120,379	35.8 (35.5–36.2)	36.4 (33.1–39.6)	35.8 (35.4–36.1)
2	115,397	32.6 (32.3–32.9)	30.8 (27.3–34.3)	32.8 (32.4–33.1)
3	47,259	12.5 (12.3–12.8)	8.1 (5.9–10.2)	12.7 (12.4–12.9)

a The number of healthy behaviors (ie, consumes ≥1 fruit and ≥1 vegetable daily, meets weekly aerobic physical activity recommendations, has a body mass index <25 kg/m^2^) was computed for each respondent by summing the number of individual healthy behaviors. Only respondents who provided data for all 3 healthy behaviors were included in the analysis.

b Unweighted number of respondents.

c Estimates are weighted and age-adjusted to the 2000 US standard population.

## Discussion

We found that, compared with adults without HOS, adults with HOS had lower odds of consuming 1 or more fruit and 1 or more vegetable daily and meeting weekly aerobic physical activity recommendations. Furthermore, adults with HOS had higher odds of not engaging in any healthy behavior and lower odds of engaging in 2 or 3 healthy behaviors than adults without HOS. These results highlight disparities in healthy behaviors between adults with and without HOS, which is concerning for stroke survivors who already have increased cardiometabolic risk.

Despite recommendations for people with HOS to engage in healthy behaviors to reduce risk of chronic disease and secondary conditions, little is known about the prevalence of engaging in healthy behaviors in this population or about weight loss interventions in people with HOS. No study with weight loss as the primary outcome has been published in people with HOS. Rather, interventions intended to promote health or manage risk factors have tracked weight as a secondary outcome. Rimmer et al demonstrated a modest weight loss of 1.27 kg after a 12-week, group-based intervention that consisted of exercise, nutrition education, and support for healthy behaviors change (10). Similarly, Joubert et al demonstrated a 0.5 reduction in BMI (equivalent to a loss of 0.68 kg in a person that weighs 90.7 kg and is 1.75 m tall) in a sample of adults with HOS who received telephone support from a nurse coordinator in advance of regularly scheduled visits with their primary care physician at 3, 6, 9, and 12 months after stroke (9). In both studies, weight loss occurred among stroke survivors with overweight or obesity in the context of interventions addressing lifestyle behaviors, including physical activity and nutritional intake. Furthermore, improvements in blood pressure and cholesterol were also observed. These studies demonstrate that weight loss can occur in conjunction with decreased cardiometabolic risk in overweight or obese people with HOS through modification of lifestyle behaviors. This is important because obesity increases cardiometabolic risk (8), which is present in 64% of adults with HOS as we report here.

Similarly, little is known about fruit and vegetable consumption in people with HOS. Studies that examine nutrition in people with HOS often investigate inadequate nutritional intake and have demonstrated that adults with dysphagia as well as community-dwelling adults with minor functional disability have decreased energy intake and protein consumption compared with adults without HOS (17–19). Surprisingly, fruit and vegetable consumption has received little attention among people with HOS. Using data from 388 stroke survivors obtained from the National Health and Nutrition Examination Survey between 1988 and 1994, Towfighi et al estimated that 98.1% of adult stroke survivors consume at least 1 serving of fruits or vegetables daily (11). This estimated prevalence is much higher than the 45.2% reported in this study, which may be explained by the difference in the number of study respondents with HOS (n = 388 vs n = 18,269) and because Towfighi et al measured fruit *or* vegetable consumption rather than fruit *and* vegetable consumption. Although our measure of fruit and vegetable consumption provides only a snapshot of one’s dietary intake, it is an important measure for at least 2 reasons. First, fruit and vegetable consumption is a known protector against stroke, where as few as 3 servings can reduce risk of stroke by 11% (20). Second, measuring low fruit and vegetable consumption is important for identifying individuals at risk for underconsumption of important nutrients that protect against chronic conditions (12). As it relates to this study, 54.8% of adults with HOS and 48% of adults without HOS reported consuming less than 1 fruit and 1 vegetable daily, which places these individuals at increased nutritional risk.

In our study, adults with HOS had 28% lower odds of meeting weekly aerobic physical activity recommendations compared with adults without HOS. These findings mirror many study results showing that physical activity is lower in people with HOS. In a recent systematic review, English et al reported that daily step counts among people with HOS were less than half those of age-matched controls, but studies reporting activity intensity were sparse (21). More recently, 2 cohort studies reported an average of 4.9 (standard deviation, 5.8) minutes (22) and 66 (standard deviation, 68) minutes (23) of moderate-to-vigorous physical activity daily among community-dwelling adults with HOS, with respective samples sizes of 37 and 25. Given the variability in minutes of physical activity between studies and standard deviations within studies, it is apparent that physical activity varies among individuals with HOS such that some individuals are very active while others are not, and that HOS by itself is not the only limiter of physical activity. In our study, 42.5% of adults with HOS reported meeting weekly aerobic physical activity recommendations, which is encouraging. The high prevalence was likely due to the inclusion on the BRFSS of leisure and household physical activity, in addition to exercise, in its calculation of aerobic physical activity.

Interventions for secondary stroke risk management have been tested, but outcomes related to changes in weight, fruit and vegetable intake, and physical activity have demonstrated limited effectiveness (24). Many of the tested interventions have been limited in duration and intensity, with many lasting less than 3 months and consisting of only 6 to 12 visits. In contrast, guidelines for the management of overweight and obesity in adults advise that lifestyle modification programs are most effective when 14 or more visits occur over 6 months or more and when caloric intake and physical activity are modified through use of behavior change strategies (14). Failure to follow these guidelines may explain why previous secondary stroke prevention programs have demonstrated limited effectiveness, whereas programs such as the Diabetes Prevention Program (2) and the Stenting versus Aggressive Medical Management for Preventing Recurrent Stroke in Intracranial Stenosis (SAMMPRIS) trial (3) have demonstrated much greater effectiveness. Because adjusted odds of not having any healthy behavior was higher and adjusted odds of 2 or 3 healthy behaviors were lower among adults with HOS in our study, adults with HOS would likely benefit from lifestyle behavior modification programs that are consistent with recommended guidelines for weight loss in terms of content, duration, and intensity.

Our study has several limitations. First, BRFSS is a self-report survey, so responses are subject to reporting bias; despite this limitation, BRFSS collects data from US adults in each state and territory, which are weighted to match known sample distributions and demographic characteristics, for disease surveillance and public health promotion. Second, BRFSS is a telephone survey conducted among noninstitutionalized, US adults; therefore, reports from individuals not living in the community or respondents with HOS whose cognitive or physical limitations interfered with their ability to participate in the survey may not be represented. Additionally, BRFSS does not collect information on stroke characteristics (eg, time since stroke, number of strokes, stroke severity), so examination of healthy behaviors among stroke subpopulations was not possible. Third, the cross-sectional nature of the study prevents determination of causality among healthy behaviors, HOS status, and demographic characteristics. Despite these limitations, reliability and validity of BRFSS questions and methodology are adequate (25). Furthermore, because BRSSS is a state-based survey conducted in all 50 states, the District of Columbia, and 3 US territories, the large sample size allowed for prevalence estimation of selected healthy behaviors among US adults with and without HOS.

Compared with adults without HOS, adults with HOS are less likely to consume at least 1 fruit and 1 vegetable daily, meet weekly aerobic physical activity recommendations, and engage in more than 1 healthy behavior. Engaging in healthy behaviors is important, because they reduce risk for recurrent stroke, development of comorbid conditions, and premature death, which is particularly important for stroke survivors, who are already at increased risk for these conditions as a result of previous stroke. Lifestyle interventions in this population are lacking but have potential to improve health and health-related quality of life. To this end, more research and lifestyle interventions are needed to address these disparities in healthy behaviors in adults with HOS.
